# Utility of susceptibility-weighted imaging in Parkinson’s disease and atypical Parkinsonian disorders

**DOI:** 10.1186/s40035-016-0064-2

**Published:** 2016-10-07

**Authors:** Zhibin Wang, Xiao-Guang Luo, Chao Gao

**Affiliations:** 1 Neurology Department, The First Affiliated Hospital of China Medical University, 155# Nanjing Bei Street Heping District, Shenyang, 110001 People’s Republic of China; 2 Neurology Department, Ruijin Hospital, Shanghai Jiaotong University School of Medicine, Ruijin 2nd Road 197, Shanghai, 200025 People’s Republic of China

**Keywords:** Parkinson’s disease, Multiple system atrophy Parkinsonian predominant type, Progressive supranuclear palsy, Susceptibility-weighted imaging, Iron deposition, Biomarker

## Abstract

In the clinic, the diagnosis of Parkinson’s disease (PD) largely depends on clinicians’ experience. When the diagnosis is made, approximately 80% of dopaminergic cells in the substantia nigra (SN) have been lost. Additionally, it is rather challenging to differentiate PD from atypical parkinsonian disorders (APD). Clinially-available 3T conventional MRI contributes little to solve these problems. The pathologic alterations of parkinsonism show abnormal brain iron deposition, and therefore susceptibility-weighted imaging (SWI), which is sensitive to iron concentration, has been applied to find iron-related lesions for the diagnosis and differentiation of PD in recent decades. Until now, the majority of research has revealed that in SWI the signal intensity changes in deep brain nuclei, such as the SN, the putamen (PUT), the globus pallidus (GP), the thalamus (TH), the red nucleus (RN) and the caudate nucleus (CN), thereby raising the possibility of early diagnosis and differentiation. Furthermore, the signal changes in SN, PUT and TH sub-regions may settle the issues with higher accuracy. In this article, we review the brain iron deposition of PD, MSA-P and PSP in SWI in the hope of exhibiting a profile of SWI features in PD, MSA and PSP and its clinical values.

## Background

Parkinson’s disease (PD) is characterized by resting tremor, rigidity, bradykinesia and postural instability accompanied by non-motor symptoms [1]. The criteria for PD diagnosis largely relay on clinicians’ experience, and an accurate diagnosis often needs 3 to 5 years of follow-up. When PD is diagnosed, approximately 80% of dopaminergic cells in the substantia nigra pars compacta (SNc) have been lost [2]. Researches miss the opportunity for unraveling the mechanism of PD in the early stage to develop disease-modifying therapy which is deemed to prevent the disease progression or complications, though no such therapy exists at present [3]. On the other hand, atypical parkinsonian disorders (APD) are a group of heterogeneous neurodegenerative diseases including multiple system atrophy parkinsonian predominant type (MSA-P), progressive supranuclear palsy (PSP), and others. However, in the early stage of parkinsonism, PD and APD often show similar symptoms that are extremely difficult to distinguish, even for experienced neurologists [4]. Thus, it is critical to find the biomarkers for the diagnosis and differentiation of PD in the early stage. In the clinic, the biomarkers detected by positron emission tomography (PET) and single photon emission computed tomography (SPECT) can directly visualize the loss of dopaminergic cells [5, 6]. However, MRI is less expensive, non-invasive and avoids the radiation of radiotracers compared with PET and SPECT. During the past decades, a number of imaging signs were found by conventional MRI such as the “swallow tail sign”, the pontine atrophy, the “hummingbird sign”, etc. Although the specificities of these signs are high, the sensitivities are highly magnetic-intensity dependent [7–9]. The ultra-high field MRI is not wildly accepted in clinic as the results of the high expense and potential security problems.

The most ideal biomarkers must be involved in the pathologic changes of PD and APD such that the potential biomarkers can indicate the underlying pathologic processes. The abnormal iron deposition in the deep brain nuclei in parkinsonism was first described by Lhermitte et al. in 1924 [10]. Most studies have discovered that iron increases consistently in SN of PD, and the iron content is associated with disease severity [11]. Recently, abnormal iron deposition was also found in APD such as MSA-P, PSP and others [12–15]. Interestingly, the regions that are rich in iron among neurodegenerative diseases vary from each other [15, 16]. Iron may play a key role in the neuropathology of neurodegenerative diseases [12]. Therefore, the iron concentration and iron distribution in deep brain nuclei may work as promising biomarkers in PD and APD. Also, iron can change the magnetic susceptibility of tissues where it deposits. Susceptibility-weighted imaging (SWI), a novel MRI technique, is sensitive to magnetic susceptibility of tissue, and thus can detect the iron-related information of neurodegenerative parkinsonism working as a promising detecting tool in PD and APD [16, 17]. Here we review about brain iron deposition of PD, MSA-P and PSP in SWI in the hope of exhibiting a profile of SWI feature in PD, MSA and PSP and its clinical values.

## Susceptibility-weighted imaging

**Fig 1:**
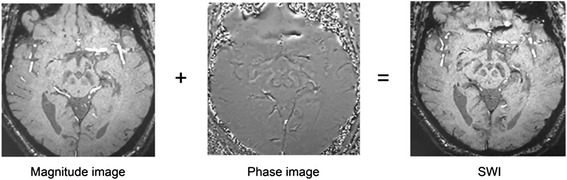
A SW image is developed from the combination of a magnitude image and a phase image

SWI exploits the differences in magnetic susceptibility of tissues, which describes the magnetic response of tissues placed in an external magnetic field, to develop an enhanced image contrast for conventional MRI [18–20]. By applying a gradient-recalled echo (GRE) sequence with relatively long echo time (TE), a SW image combines a phase image with a magnitude image under high-intensity magnetic field such as 3T and 7T, to add the magnetic susceptibility information to the structure of the brain in situ (as shown in Fig. 1) [19]. The high-intensity field ensures a high spatial resolution and contrast-to-noise ratio for the further study of detailed structures in the brain [21]. Image phase variations reflect the static magnetic field inhomogeneities, which are influenced by a macroscopic effect and a microscopic effect [19, 22]. The macroscopic effect, also called the geometry effect, is that the configuration of tissues, such as white matter tract, capillary beds, the interstitial space and others, distorts the homogeneity of the local field [19, 22, 23]. The microscopic effect is described as the homogeneity of the local field being distorted by substances with different magnetic susceptibility [18, 19, 22, 24]. Thus, the variances in the magnetic susceptibility of tissues are derived from both the geometry of the tissue and the substances’ reaction to the applied field.

According to the reaction to the applied field, the substances can be classified into paramagnetic and diamagnetic. The paramagnetic substances mainly include ferritin containing ferric iron, deoxygenated hemoglobin, and ceruloplasmin, while the diamagnetic ones include myelin, calcium, and oxygenated hemoglobin [23]. Generally, the grey matter is paramagnetic because of the iron, and the white matter is diamagnetic due to the myelin [25, 26]. In comparison, iron has the highest concentration in the deep grey nuclei, while the other substances are relatively minimal [12]. Abnormal iron deposition patterns were found in the brain of patients with parkinsonism. Excess iron can cause damage to neuron through free radical production [13, 27, 28]. Therefore, iron is thought to play a key role in the pathogenesis of neurodegenerative parkinsonism [12, 13, 28, 29]. The abnormal iron deposition results in changed iron distribution and concentration in the brain, and thereby changing the susceptibility of tissue. SWI is able to identify the susceptibility alteration through recording the phase changes caused by the iron deposition through the multiplication of phase image with magnitude image for several times, which enables the increasing of phase contrast and indirect evaluation of iron content in parkinsonism [30–33]. What’s more, the sensitivity of SWI is higher and the error under the same signal-to-noise ratio is lower compare with T2*-weighted imaging, often considered the gold standard for the evaluation of brain iron content [34–38]. Thus, SWI is reliable for the clinical usage of evaluating the iron content of deep brain nuclei. However, SWI has drawbacks as well. The phase is not a local physical property, because the phase of a certain region is affected not only by the susceptibility of this region, but also by that of the surrounding areas [23, 26, 39]. It means that the phase image reflects comprehensive information about susceptibility changes. Moreover, a process called convolution in the formation of phase image makes it very difficult to distinguish paramagnetic iron from diamagnetic calcium [23]. Furthermore, the reproducibility is low, because the phase can be affected by the orientation of structures relative to the applied field [40].

To solve the problems of SWI, quantitative susceptibility mapping (QSM) is designed to quantify the iron content through measuring the susceptibility of certain areas directly [23, 39–43]. QSM is developed from SWI by solving the ill-posed inverse effect-to-source problem. Because the susceptibility is an intrinsic property of tissue, QSM can produce more precise image avoiding the non-locality of phase, and can differentiate iron from calcium [23]. Moreover, QSM can avoid the effect of the orientation relative to the applied field, due to the susceptibility value in QSM is isotropic [26]. What’s more, QSM shows a higher reproducibility compared with R2* mapping to measure iron content [40]. Although this technique may be useful for assessing iron in deep structure with high iron content, the diamagnetic myelin level in white matter may impact the measurement. Little research has been conducted about the application of QSM for the diagnosis and differentiation of parkinsonism [42–44]. Therefore, QSM has a very promising future to study the parkinsonism-related iron deposition [36].

## Brain iron deposition

### Brain iron accumulation in the physiologic state

#### The brain iron distribution is uneven

The iron levels of basal ganglia are high [34, 45, 46]. Histological studies have shown that normally the caudate nucleus (CN), the putamen (PUT), the globus palidus (GP), the red nucleus (RN), and the substantia nigra (SN) are rich in iron, while the iron content of cortex is relatively low [45, 46]. In the cortex, the motor cortex (MC) is richest in iron, while the prefrontal and temporal cortices are the poorest [45]. Although the exact order of the iron level of deep nuclei does not reach a consensus between postmortem studies, those studies imply the heterogeneous distribution of brain iron in normal aging [45, 46].

Even within the same deep brain nuclei, the iron distribution is uneven. Histological studies reveal that the SN pars reticulata (SNr) in the ventral SN is rich in iron, while the SNc in the dorsol SN is poor [47, 48]. Zecca et al. reported that the neuromelanin is the major iron-stored place in the SN neurons [49]. According to these facts, it is concluded that physiologically, iron deposition is heterogeneous, even in the same deep brain nuclei like SN.

#### The speed of brain iron deposition is uneven

A bunch of studies have approved that the speed of iron deposition across the brain is not even. In SN and GP, the iron concentration grows quickly in the early 20 years of life, then more gradually, and stops after 30 years of age [45, 49]. For CN and PUT, the maximal speed of iron accumulation was during the first 50 to 60 years old of life [45]. In some areas of the brain, iron deposition occurs through the whole life span. Interestingly, the iron content of the medulla oblongata is low and does not increase with advancing of age [45, 46].

#### The function of the brain iron

Iron functioning in the brain mainly exists in the forms of hemoglobin, iron-containing enzyme and non-haemin iron [13, 28, 45]. The iron involved in normal aging and neurodegenerative parkinsonism belongs to the non-haemin which is a cofactor of several enzymes that are associated with myelin formation and neurotransmitter production [13, 27–29, 45, 50, 51]. The period of the fastest iron deposition coincides with when the myelin of the brain forms the fastest in the early stage of life [12, 29, 45]. There is other evidence that iron deficiency can impair neural development, behavioral and cognitive function probably via damage to the myelin formation and neurotransmitter production [28].

### Brain iron deposition in neurodegenerative parkinsonism

#### Brain iron accumulation in PD

Pathological and MRI studies indicate that the SN is the most relevant area of the brain in PD [17, 49, 52–55]. The iron concentration of the SN on the affected side of PD is approximately 80% more than that in healthy controls (HC), while overall iron contents in the brain between PD and HC are close [2, 31]. The reason for this phenomenon is unclear, but the elevated iron level of SN may relate to the localized pathogenesis such as permeability changes of the blood brain barrier (BBB), inflammation state, gene mutation-induced abnormal protein function in iron storage and transport, etc. [56–58] Furthermore, excess iron can cause cell death through reactive oxygen species derived from Fenton’s reaction by which iron catalyzes hydrogen peroxide [11–13, 27, 29]. Notably, an insight into the relationship between the ferric (III) or ferrous (II) state of iron and alpha-synuclein enables a better understanding of the pathogenesis of PD. Fe (II) takes part in the Fenton reaction producing hydrogen peroxide, and then Fe (II) is oxidized to Fe (III). Levin et al. reported that alpha-synuclein aggregation is independent of oxidizing agents, while is highly correlated with the amount of Fe (III) [59]. This is in line with the fact that the Fe2+/Fe3+ ratio shift to the Fe3+ in PD [59, 60]. Iron chelators can protect neurons from death in vitro, and PD patients may benefit from the decrease in iron level [12, 61, 62]. Some researchers believe that a localized, elevated iron level is caused by the iron-chelated neuromelanin that is released from dead neurones injured by aggregation of alpha-synuclein [54]. In this case, the increased extracellular neuromelanin is the cause of elevated iron level. In contrary, others suggested that the iron level increased primarily, and then neuromelanin increased as a compensatory factor to chelate redundant iron [11]. It is still debatable whether abnormal iron deposition is the primary cause of neuropathology or just an epiphenomenon [12, 13].

#### Brain iron accumulation in APD

MSA and PSP are the most common APD and clinically the most important differential diagnosis for PD [48]. Autopsy research suggested that brownish discoloration of deep brain nuclei relates to iron deposition [48, 63]. In MSA, discoloration and atrophy of posteriolateral PUT was remarkable compared with PD and PSP in autopsy [48, 63, 64]. However, neither discoloration nor atrophy of RN, dendate nucleus (DN) and subthalamic nucleus (STN) were found in MSA [48, 63, 64]. In PSP, remarkable atrophy and discoloration were revealed in cerebellar WM and STN [65–68]. Both MSA and PSP showed various degrees of the atrophy of SN, GP, TH and CN [48, 63–66, 68]. More researches are needed to further specify the spatial distribution of iron deposition in APD.

## SWI in Parkinson’s disease and atypical parkinsonian disorders

### SWI in the diagnosis of Parkinson’s disease

The majority of research approved that SWI was feasible to indirectly quantify the iron content of different regions of brain through comparing the phase values in SWI which was highly correlated with the iron content [17, 19, 22, 24]. Thus, the comparison of iron content in SWI is conducted by comparing the phase value indirectly rather than by comparing the iron content directly like QSM. There are various forms of iron deposition patterns of PD imaged by SWI among the researches. Some researches supported that iron deposition patterns in SWI can distinguish PD from HC. Jiuquan Zhang et al. reported that the iron concentration was elevated significantly only in the SN of PD compared with HC in SWI [17]. The iron contents of the SNc, CN and RN in PD were significantly higher than those in HC in a SWI study by Wei Zhang et al. [33]. Also, Wu et al. demonstrated the iron accumulation in the SN, RN, CN, PUT, and GP of PD was more remarkable than that of HC [69]. An elevated iron level of the SN was common in PD among SWI research, because the SN is the most pathologically relevant site of PD and become atrophy and brownish discoloration in autopsy [48, 67, 70]. Notably, Dashtipour et al. did not find remarkably increased iron content of SN in PD, and it may be explained by the small sample size [31]. However, the discrepancy of iron accumulation in other nuclei, such as RN, CN, PUT, and GP, in SWI is unclear. One possible reason is that deep brain nuclei are pathologically involved simultaneously with different degrees of iron accumulation [70]. In addition, different iron deposition patterns may relate to disease progression [31]. Furthermore, research has demonstrated that the iron content of the SN is inversely correlated with the severity of PD as measured by UPDRS-motor score and H-Y stage [17, 30, 48], while no correlations were found between the iron content of the SN and the duration, progression, prognosis and levodopa response of PD [17, 31, 33, 69, 71, 72]. Even though with the heterogeneity of iron deposition speed and distribution in the whole disease process, SWI still fails to characterize specific clinical features of PD. For instance, SWI cannot detect the difference between earlier-onset and later-onset PD patients [17]. Neither could SWI show difference between the early and intermediate/advanced stages of PD [69]. Mechanisms, such as gene mutation, alteration of the BBB, and inflammation, may underlie the speed, onset and spatial distribution of iron deposition [12, 29, 50]. Further studies are needed to figure out whether there are correlations between iron content of SN and specific clinical features.

**Fig 2:**
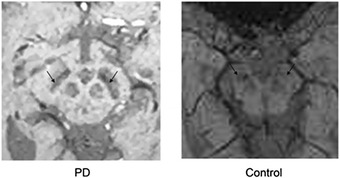
Nigrosome 1 of three-layered structure disappears in PD patients, while it exists in health controls (which are pointed out by black arrows)

In recent years, a novel imaging biomarker called nigrosome 1, which is the sub-region of the SN, has been extensively studied by researchers. According to the immuno-staining of calbindin that can bind to calcium, the SNc is subdivided into nigrosome (caldbindin-poor) and nigral matrix (caldbindin-rich). Nigrosome 1 is the largest nigrosome containing the biggest group of dopaminergic cells and is affected in almost every PD patient [73]. It was reported that 7T MRI could visualize the three-layered structure of SN and could distinguish patients with PD from HC with both high sensitivity and specificity [14, 74, 75]. In 3T SWI, nigrosome 1 also shows dorsolateral hyperintensity of SN in HC, and disappears in PD with 100% sensitivity and high specificity (shown in Fig. 2) [47, 73]. These evidences suggested that 3T SWI is a reliable tool for the visualization of nigrosome 1 and the diagnosis of PD. However, in some studies nigrosome 1 hyperintensity also disappears in MSA-P and PSP in SWI [14, 53]. Therefore, nigrosome 1 is a safe biomarker for neurodegenerative parkinsonism rather than PD.

### SWI in the differentiation of PD from APD

#### The iron deposition patterns of MSA-P imaged by SWI

At present, the most promising signs in SWI that characterize MSA-P focus on signal changes in PUT due to its remarkably high iron level demonstrated by autopsy [32, 48, 63, 76]. As the mechanism of SWI, the signal intensity of certain region is decreased when paramagnetic iron accumulates. Researchers designed a visual scale of the PUT hypointensity for visual differentiation [71, 76, 77]. The visual scales contain a set of standard images of putaminal hypointensity graded from 0 to 3. The higher the grade reaches, the lower the signal intensity is [71, 76, 77]. Grade 3 hypointensity of the posterior PUT was reported to discriminate MSA-P from PD [77]. This finding was consistent with the iron deposition pattern of MSA-P in autopsy that the iron content of posterior PUT in MSA-P was remarkably higher than that in PD [48, 63, 64]. Some researchers even suggested more detailed criteria for differentiation. For example, Wang et al. reported that when the PUT was sub-divided into 4 regions (lower inner, lower outer, upper inner and upper outer), the lower inner part of the PUT performed the best on receiver operating characteristic curve to distinguish MSA-P from PD [72]. Except for the studies about the iron levels in the local areas of PUT, Han et al. found that the topography of iron deposition of the PUT showing posteriolateral-to-anteriomedial ascending signal intensity, was highly specific for MSA-P patients, even in the early stage without obvious clinical symptoms [32]. Although the aforementioned biomarkers are still controversial and need further validation, they are still by far the most promising biomarkers and are clinically available. It was even suggested by Yoon et al. that SWI was potentially able to replace PET in the diagnosis of MSA-P because in PUT there was positive correlation between a low metabolism rate in PET and the low signal intensity in SWI [16]. However, Kwon et al. found no correlation between the metabolism rate in PET and the signal intensity in SWI [78]. The potential that SWI indicates the dysfunction of dopaminergic neurons imaged by PET needs further studies.

Other deep nuclei, such as the CN and pulvinar TH (PT), were also studied. Meijer et al. reported that the iron content of the CN on the affected side of MSA-P was remarkably higher than that of PD in SWI [77]. By contrast, Wang et al. demonstrated that the iron content of the CN cannot distinguish MSA-P from PD in SWI [72]. On autopsy, neuronal loss of the CN correlated with iron deposition is common and severe in MSA-P but unusual and mild in PD [12, 48]. The discrepancies between different studies may come from the variety of inclusion criteria, with patients at different MSA-P stages. Only a few study investigated about PT. Wang et al. found that the higher iron content of the PT in MSA-P, but failed to show statistical significance between groups [72]. For CN and PT, more researches are warranted to validate their potentials as biomarkers.

#### The iron deposition patterns of PSP imaged by SWI

There are different iron deposition patterns of PSP in SWI because of various forms of neuropathological processes [65]. Meijer et al. reported that elevated iron levels of the RN and dentate nucleus (DN) on the affected side could distinguish PSP from PD [77]. These findings are consistent with the pathological results of Dickson that only the RN and DN were damaged consistently and severely in PSP, but were spared in PD and MSA [48]. Gupta et al. found that increased iron content of the RN and the PUT was able to differentiate PSP from MSA-P and PD [71]. Notably, the iron level of the PUT is suggested to be the biomarker for the diagnosis of MSA-P in many studies [32, 48, 63, 76]. One possible speculation for this controversy is that local iron content and specific deposition patterns such as dense iron deposition in lower outer part of PUT, are more specific than overall content in MSA-P, while the overall iron content of PUT is a characteristic feature for PSP [71, 76, 77]. In addition, Han et al. demonstrated that elevated iron contents of GP and TH were the most valuable biomarkers in SWI to differentiate PSP from MSA-P and PD [32]. From neuropathological studies, neuronal loss of GP and TH is more severe in PSP than in MSA, but spared in PD [48, 63, 66]. However, due to the diverse results, consensus regarding the features of SWI imaging for PSP is hard to reach and further studies aiming at finding SWI biomarkers should consider the disease stage and combine the SWI results with iron-related neuropathology.

## The drawbacks among SWI research

SWI is a promising biomarker that provides more information for early and differential diagnosis of parkinsonism, however present studies have several drawbacks that need to be addressed in the future. (1) Few research combined the SWI results of parkinsonism with pathological investigations [32, 71]. (2) Most of patients involved in studies have received levodopa replacement therapy, which may change the iron deposition pattern in SWI [17]. (3) Most of the studies are retrospective [72], and the follow-up of a few prospective studies are relatively short [8]. (4) The difference in scan parameters of SWI such as slice thickness, matrix size, etc., should be considered to interpret the discrepancy of results [17, 69]. Also, standardization for regions of interest drawn by hand and uniformed image analysis should be applied [33].

## Conclusion

SWI characterizes brain iron deposition patterns of PD to illustrate the iron-related pathologic alterations in vivo and compensates for some drawbacks of routine MRI. Many researches have confirmed that SWI is a promising tool for the diagnosis and differential diagnosis of PD through discovering iron-related biomarkers, and more accessible in the clinic. Further studies should take the stages of neurodegenerative parkinsonism into consideration to acquire better correlations between SWI findings and neuropathologic results. With the underling pathological procedures illustrated by SWI, it will be possible to diagnose PD in the early stage and differentiate PD from APD.

## Abbreviations

APD: Atypical parkinsonian disorders
BBB: Blood brain barrier
CN: Caudate nucleus
DN: Dentate nucleus
GM: Grey matter
GP: Globus pallidus
HC: Healthy controls
LC: Locus coeruleus
MC: Motor cortex
MSA-P: Multiple system atrophy parkinsonian predominant type
PD: Parkinson‟s disease
PSP: Progressive supranuclear palsy
PT: Pulvinar thalamus
PUT: Putamen
RN: Red nucleus
SN: Substantia nigra
SNc: SN pars compacta
SNr: SN pars reticulate
STN: Subthalamic nucleus
SWI: Susceptibility-weighted imaging
TH: Thalamus
WM: White matter
